# Effectiveness of the baby‐friendly community initiative on exclusive breastfeeding in Kenya

**DOI:** 10.1111/mcn.13142

**Published:** 2021-02-02

**Authors:** Elizabeth W. Kimani‐Murage, Judith Kimiywe, Antonina N. Mutoro, Calistus Wilunda, Frederick Murunga Wekesah, Peter Muriuki, Bonaventure M. Mwangi, Betty Mogesi Samburu, Nyovani Janet Madise, Stephen T. McGarvey, Paula L. Griffiths

**Affiliations:** ^1^ Maternal and Child Wellbeing Unit African Population and Health Research Center Nairobi Kenya; ^2^ International Health Institute, School of Medicine, Dentistry and Nursing, College of Medical, Veterinary & Life of Public Health Brown University Providence RI USA; ^3^ Department of Food, Nutrition and Dietetics Kenyatta University Nairobi Kenya; ^4^ Human Nutrition University of Glasgow Glasgow UK; ^5^ Formerly Division of Nutrition and Dietetics Ministry of Health in Kenya Nairobi Kenya; ^6^ Nutrition Section United Nations Children's Fund (UNICEF) Kenya Country office Nairobi Kenya; ^7^ Research and Development Policy African Institute for Development Policy Lilongwe Malawi; ^8^ School of Sport, Exercise and Health Sciences Loughborough University Loughborough UK; ^9^ School of Clinical Medicine University of the Witwatersrand Johannesburg South Africa

**Keywords:** baby‐friendly community initiative, child nutrition, cluster randomized trial, exclusive breastfeeding, infant feeding practices, Kenya, rural

## Abstract

The baby‐friendly hospital initiative (BFHI) promotes exclusive breastfeeding (EBF) in hospitals, but this is not accessible in rural settings where mothers give birth at home, hence the need for a community intervention. We tested the effectiveness of the baby‐friendly community initiative (BFCI) on EBF in rural Kenya. This cluster randomized study was conducted in 13 community units in Koibatek sub‐county. Pregnant women aged 15–49 years were recruited and followed up until their children were 6 months old. Mothers in the intervention group received standard maternal, infant and young child nutrition counselling, support from trained community health volunteers, health professionals and community and mother support groups, whereas those in the control group received standard counselling only. Data on breastfeeding practices were collected longitudinally. The probability of EBF up to 6 months of age and the restricted mean survival time difference were estimated. A total of 823 (intervention group *n* = 351) pregnant women were recruited. Compared with children in the control group, children in the intervention group were more likely to exclusively breastfeed for 6 months (79.2% vs. 54.5%; *P* < .05). Children in the intervention group were also exclusively breastfed for a longer time, mean difference (95% confidence interval [CI]) 0.62 months (0.38, 0.85; *P* < .001). The BFCI implemented within the existing health system and including community and mother support groups led to a significant increase in EBF in a rural Kenyan setting. This intervention has the potential to improve EBF rates in similar settings.

**Key messages**

- Exclusive breastfeeding rates can be increased when hospital‐based interventions are complemented with community‐based interventions that build on the existing healthcare infrastructure in rural Kenya.
- This intervention has good potential for scale‐up because it relies on the health system to train and support community health volunteers as well as community and mother support groups.
- Community health volunteers played a vital role in implementation of this intervention. Innovative solutions such as the incorporation of income‐generating activities for the CHVs can be used to ensure sustainability.

## 1 INTRODUCTION

Exclusive breastfeeding (EBF) for the first 6 months of a child's life and continued breastfeeding thereafter for up to 2 years, with timely introduction of appropriate complementary feeding at 6 months, is essential for optimal child growth and development. Numerous health, financial, social and economic benefits of EBF have been documented (Victora et al., 2016). Despite the documented benefits of EBF to children and their families, EBF rates remain low in most parts of the world. The 2019 Global Breastfeeding Scorecard reported that 41% of children under 6 months are exclusively breastfed (WHO & UNICEF, 2019). In Kenya, EBF rates are at 61%, and although this is a significant increase from 2008, there is a still a need for further improvement (Kenya National Bureau of Statistics, 2010, 2015). However, it is important to note that the rates are based on self‐report and are therefore subject to recall and report bias.

Various initiatives, such as the baby‐friendly hospital initiative (BFHI), have been put in place to strengthen maternity services to promote EBF for the first 6 months of life and adequate and safe complementary feeding with continued breastfeeding up to 2 years of age or beyond (WHO, 2018). The BFHI was developed in the 1990s by the World Health Organization (WHO) and UNICEF as a global programme to improve EBF in maternity facilities. BFHI consists of 10 steps, which summarize a package of policies and procedures required to support breastfeeding. Studies have shown that the initiative is cost‐effective and has a short‐term positive impact on breastfeeding outcomes at facility level (Kim et al., 2018; Pérez‐Escamilla et al., 2016). For sustainability, however, the 10th step of the BFHI, which is related to community promotion of breastfeeding, needs to be scaled up hence the development of the baby‐friendly community initiative (BFCI), which aims to promote optimal maternal, infant and young child nutrition (MIYCN) at the community level.

The BFCI package was adapted for implementation by the Ministry of Health in Kenya, where 39% of mothers give birth at home (Kenya National Bureau of Statistics, 2015; Ministry of Health, 2016). The BFCI involves an eight‐step plan, which extends follow‐up and care of the mother/child from the health facility to the community, so as to provide women with a comprehensive support system to improve breastfeeding and other MIYCN practices (Ministry of Health, 2016). It is implemented within the community health strategy (CHS), a programme established by the Kenyan government in 2007 (Ministry of Health, 2007). The strategy uses a vast network of community health volunteers (CHVs) to promote healthcare at the community level. It also establishes a basic care unit known as a community unit (CU) with a population of approximately 5,000 people (WHO, 2017). The CHVs are trained on various aspects of primary healthcare including but not limited to maternal and child health and sexual and reproductive health. Each CHV provides services to between 10 and 100 households (WHO, 2017).

The BFCI relies heavily on counselling of mothers by CHVs, who are empowered and facilitated with knowledge, skills and job aids to provide home‐based counselling to pregnant women and mothers with infants and young children. The initiative also creates a strong link between healthcare service providers at facility level and the community.

Although anecdotal evidence points to the BFCI being effective in promoting EBF, there is generally little empirical evidence on which to base this claim (Cattaneo et al., 2016; Kavle et al., 2019). Assessing the effectiveness of implementing the BFCI on EBF in the first 6 months using a cluster randomized trial was therefore necessary. Cluster randomization was preferred over individual‐level randomization to minimize contamination of messages between individuals and for pragmatic purposes in case of future scale‐up of the intervention, which would rely on the CU administrative system to implement the programme.

## 2 METHODS

### 2.1 Study setting

In 2014, the median duration of EBF in the Rift Valley was 3.1 months and half of women delivered at home (Kenya National Bureau of Statistics, 2015). The study was therefore conducted in Koibatek/Eldama Ravine sub‐county, one of the six sub‐counties in Baringo County located in the North Rift region of Kenya. The total population of Koibatek sub‐county in 2014 was 125,637 of which 30, 203 were women of reproductive age and 4,799 were children under 1 year (Kenya National Bureau of Statistics, 2019). The sub‐county is divided into four administrative divisions: Eldama Ravine, Timboroa, Esageri and Torongo. Mixed farming is widely practiced in this sub‐county, which covers 2,306 km^2^ with a population density of 55 people per km^2^.

### 2.2 Study design and randomization

This was a community‐based cluster randomized controlled trial (Trial Registration: ISRCTN03467700) whose details have been reported elsewhere (Kimani‐Murage et al., 2015). Clusters were based on CUs and were selected because any future interventions would need to map onto these administrative units to be effective. A total of 13 clusters were randomized and assigned, six into the BFCI intervention and seven in the control arm. The random sequence of allocation of the CUs to either BFCI or control group was computer‐generated and was performed by a biostatistician, who was not a member of the research team. Training of health care workers, data collectors and CHVs was conducted before the trial, which ran between April 2015 and December 2016.

### 2.3 Study population, inclusion and exclusion criteria

The trial targeted all pregnant women aged 15–49 years and their respective children who lived in Koibatek sub‐county. Recruitment of study participants took place between April 2015 and May 2016. Before commencement of the recruitment exercise, a community mobilization exercise involving community leaders was conducted to inform the community about the upcoming study in all the 13 clusters. Pregnant women were identified in the community and health facilities by CHVs and health workers, respectively. They were then recruited by the data collector. Participants were recruited as early as possible in their pregnancies in order to increase their exposure to the intervention. The women were recruited on a continuous basis until the desired sample was reached.

**FIGURE 1:**
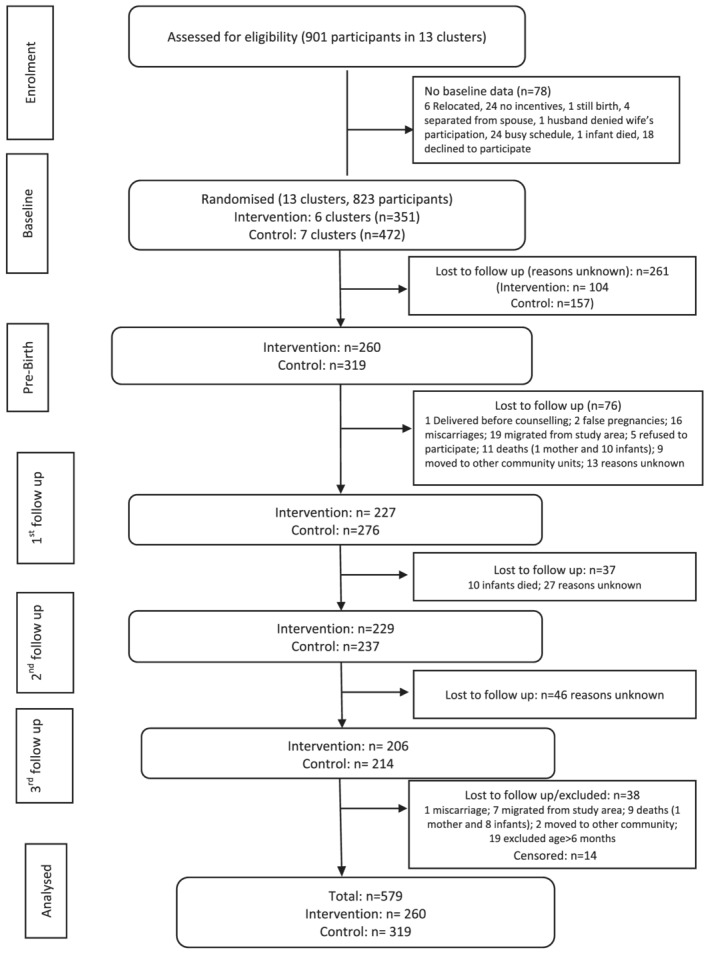
Study flow diagram. Prebirth data collected during the second half of pregnancy. Participant recruitment was on a rolling basis; therefore, recruitment stopped a few months before the study ended. ^†^Censored participants were recruited towards the end of the study and were therefore not followed up because the study ended

After initial recruitment, participants were excluded from the analysis if they declined to participate in the study, did not receive a visit by a CHV before delivery, migrated out of the study area and had a miscarriage or a still birth (Figure 1).

### 2.4 Sample size calculation

Sample size was estimated for a cluster randomized study design to be 738 mother–child pairs in 13 clusters in both the intervention and control arms. Accounting for 10% loss to follow‐up, the sample size was increased to 812, with a target to recruit 406 women in each study arm. We used a level of precision of 5% (for a two‐sided *t* test) and power of 80%. The sample size was adjusted for a design effect of 3.15, calculated based on intracluster correlation coefficient of 0.035 and an average cluster size of 62.5 and a potential loss to follow‐up of 10%. This sample size was sufficient to detect an increase in EBF between 0 and 6 months (the primary outcome) from the baseline rate of 32–50%, an 18% increase (Kimani‐Murage et al., 2015).

### 2.5 Patient and public involvement

A formative qualitative study was conducted in August 2014, in the study area to understand the local contexts and cultural factors that influence maternal nutrition, breastfeeding and other infant and young child feeding practices and community perspectives on the proposed intervention before the intervention was rolled out (Kimani‐Murage et al., 2015). The formative study involved women of reproductive age group and would be target participants in the intervention group, men and other community members (grandmothers, community leaders, healthcare providers and policymakers). The information generated was used to adapt the intervention to the local context, by incorporating social norms and preferences of the potential beneficiaries. The information also aided in understanding potential barriers and facilitating factors that would impact the implementation of the intervention.

Further, midline and end‐line evaluations were conducted to obtain perspectives from the participants and the community on the intervention. A series of dissemination meetings were held with different stakeholder groups at the end of the study, starting with community members. This engagement with community members informed further dissemination activities with other stakeholder groups including policymakers.

### 2.6 Training of healthcare providers

A total of 6 master trainers, accredited trainer of trainers (TOTs) working for the Ministry of Health in Kenya (MOH), participated in a 5‐day training workshop on the BFCI implementation package (Ministry of Health, 2016). Twenty‐five participants composed of the sub‐county health management team (SCHMT), health workers from the sub‐county and two community health extension workers (CHEWs) from each of the six intervention units were trained. The implementation package was developed by the MoH, adapted from the WHO/UNICEF integrated infant and young child feeding counselling course (Ministry of Health, 2016; WHO, 2016). This was followed by on‐the‐job training and mentoring on a quarterly basis to ensure proper implementation of the BFCI at the community and facility levels by the SCHMT members, specifically the Sub‐County Nutrition Officer and the Sub‐County Community Health Strategy Focal person.

CHVs, traditional birth attendants (TBAs) and healthcare providers at the community and at the lower‐level facilities including clinics, dispensaries and health centres in the intervention CUs also received training on nutrition education and counselling skills and establishment and conducting of monthly mother‐to‐mother support group (MTMSG) meetings. To motivate the CHVs and provide them with a source of income, a seed grant of $1,000 was provided to the whole CU (composed of CHVs, CHEWs and CU committee members) in all the intervention and control clusters to start an income generation project of their choice. This was complimented with a facilitated training session on income generating activities. The idea for this activity came out of the initial community consultation process, where the community and CHVs identified the difficulty that CHVs face in funding their work because of a lack of payment for the work that they undertake.

### 2.7 BFCI intervention

The BFCI package involves an eight‐step plan as shown in Table 1. The package aims at equipping primary healthcare workers with skills to empower mothers to optimally breastfeed and feed their infants and young children (Ministry of Health, 2016). The primary healthcare workers and the CHVs were provided with counselling aids that included brightly coloured cards depicting key IYCF concepts and behaviours to share with mothers, fathers and other caregivers. The package was adapted to include maternal nutrition and health counselling messages such as good maternal nutrition and early antenatal care (ANC), importance of EBF and the key processes including early initiation to breastfeeding, feeding of colostrum and attachment and positioning. Mothers were also counselled on ways of preventing mother to child transmission (PMTCT) of HIV, solving breastfeeding difficulties and obtaining family support.

**TABLE 1:** Steps in the BFCI programme in Kenya

Step	Description
Step 1	Have a written MIYCN policy summary statement that is routinely communicated to all health providers, community health volunteers and community
Step 2	Train all healthcare providers and community health volunteers in the knowledge and skills necessary to implement the MIYCN policy
Step 3	Promote optimal maternal nutrition among women and their families
Step 4	Inform all mothers and their families about the benefits of breastfeeding and risks of artificial feeding
Step 5	Support mothers to initiate breastfeeding within the first hour of birth, establish and maintain exclusive breastfeeding for first 6 months
Step 6	Encourage sustained breastfeeding beyond 6 months to 2 years or more alongside timely introduction of appropriate, adequate and safe complementary foods
Step 7	Provide a welcoming and conducive environment for breastfeeding families
Step 8	Promote collaboration between health care staff, maternal, infant and young child nutrition support groups and the local community

Abbreviations: BFCI, baby friendly community initiative; MIYCN, maternal, infant and young child nutrition.

A minimum of eight home‐based counselling sessions were offered to mothers in the intervention group by trained CHVs. The CHVs visited the pregnant women after they had enrolled. Visits occurred approximately once every month up to Week 37 of pregnancy and thereafter every 2 weeks through the first month postpartum, then once a month until the infant was 6 months old. The visits were scheduled based on the participant availability and need; that is, women who had challenges feeding their infants received more visits.

In addition, community mother support groups (CMSGs) were set up across all six units in the intervention arm, with the support and the facilitation of the CHVs and CHEWS. Roles of the CMSGs included to oversee, plan and execute community meetings on the BFCI; mobilize all community members to participate in BFCI activities; support CHEWs and nutritionist in monitoring and documenting monthly BFCI activities at the community level; and to monitor and document the MIYCN activities on a monthly basis. Apart from the pregnant women and lactating mothers, each CMSG consisted of another 9–11 members selected from the community with the help of local leaders and committee members from participating health facilities. Core members of the support group included a CHEW, nutritionist, community health committee (CHC) representative, CHVs, a local administrator (Chief or Assistant Chief), a community leader, an older woman and lead mother. Other members included religious leaders, opinion leaders and birth companions. The group met every 2 months with family members of study participants, TBAs, herbalists, political leaders like Member of the County Assembly (MCAs) and ward representatives.

Twenty‐three MTMSGs were also established in all of the six CUs in the intervention arm. Members of these support groups met every month for peer support. They consisted of pregnant and breastfeeding mothers who came together to learn about and discuss issues regarding pregnancy, infant and young child nutrition and other health related issues. Pregnant and lactating mothers in the intervention group were recruited by CHVs and the lead mothers during home visits, ANC visits and other community gatherings. Each support group had 9–15 members. When the membership exceeded 15, a new mother support group was established. In some CUs, MTMSGs also incorporated partners and spouses of the women, traditional herbalists and even grandmothers. Each mother support group had a leader known as the lead mother who worked with the CHV in facilitating group activities and acted as a link between the MTMSG, the CMSG and the health facility. The lead mother supported the CHVs in collecting BFCI data as well as identification and referral of mothers to the support group.

The control group received the standard care package, which included routine services offered to mothers and their children through the healthcare system including information materials on MIYCN, standard counselling on antenatal and postnatal care, appropriate tests during pregnancy, health facility delivery, general nutrition, hygiene and immunization. The control groups also received routine visits from CHVs as provided for within the CHS. Table 2 summarizes the services offered to the intervention and the control groups. Mothers in both the control and intervention group received MIYCN materials, which is part of the usual care offered through the health system. The MIYCN materials provided information on hygiene practices, breastfeeding and complementary feeding.

**TABLE 2:** Services and materials provided to intervention and control groups

Intervention group	Control group
BFCI training in addition to the already attained basic community health modules for CHVs	Training on basic community health modules for CHVs
Supportive supervision (scheduled regular visits to assess implementation of BFCI package) + orientation and continuous on job training and mentoring of the health workers and CHVs/CHEWs on BFCI package implementation	Supportive supervision (usual planned visits by sub‐county health management teams)
Formation of mother and community support groups, which met regularly	
Distribution of MIYCN educational materials (usual care)	Distribution of MIYCN educational Materials (usual care)

Abbreviations: BFCI, baby friendly community initiative; CHEWs, community health extension workers; CHVs, community health volunteers; MIYCN, maternal, infant and young child nutrition.

### 2.8 Outcomes

The primary outcome measure was EBF for the first 6 months. The advice and support received by mothers through the intervention was expected to lead to improved self‐efficacy with regard to breastfeeding according to WHO guidelines, resulting in improved levels of EBF in the first 6 months. Data on breastfeeding practices were collected at approximately 2, 4 and 6 months, but due to difficulties in reaching some of the participants, these ages were not strictly adhered to, hence the need to refer to these periods as first (at approximately 2 months), second (at approximately 4 months) and third (at approximately 6 months) follow‐up. Data on breastfeeding were collected through an interviewer‐administered 24‐h dietary recall questionnaire, but this questionnaire was only used during the first follow‐up. During subsequent follow‐ups, a 3‐day dietary recall was used because by omission; the translated tool did not have the 24‐h recall section. EBF was determined using two variables: (1) what foods has the child been offered over the last 24 h and (2) has the child been given solids/liquids other than breast milk. If the child was offered other foods/drinks then, the child was considered not to be exclusively breastfed. Socio‐demographic information was also collected.

Information on knowledge, attitudes and practices on MIYCN, EBF, child morbidity (diarrhoea, fever and respiratory infections) and dietary practices was also collected using a questionnaire, and child weight, length and mid‐upper arm circumference (MUAC) were taken at approximately 2, 4 and 6 months. These findings will be presented in a subsequent manuscript.

### 2.9 Data analysis

Data were analysed using Stata version 15.1. Characteristics of study participants were summarized using descriptive statistics, and Pearson's chi‐squared tests, adjusted for clustering, were used to assess differences between intervention and control groups. Some children had random missing data; for example, 243 had EBF data for only one follow‐up visit. We therefore performed both complete‐case analysis and multiple imputation to account for the missing data. Multiple imputation can perform well with even up to 50% missing observations (Kontopantelis et al., 2017). We generated 20 multiple imputed data sets using chained equations (MICE procedure in Stata). A linear regression model was used to impute child's age, and logistic regression was used to impute EBF status. The imputation models included maternal age, maternal education, maternal occupation, parity and household food security. Estimates across imputed data sets were automatically combined to obtain one set of inferential statistics.

Logistic regression models estimating odds ratios (ORs) with 95% confidence intervals (CIs) were used to assess the effect of the intervention on EBF, stratified by child's age (0–2 and 3–6 months). The probability of EBF according to study group was plotted using Kaplan–Meier survival curves, and the difference between the curves was assessed using a log rank test. Our initial plan was to use Cox regression to assess the effect of the intervention on EBF over the 6‐month follow‐up time. However, assessment of the proportional hazard assumption of the model using the scaled Schoenfeld residuals showed that the model violated this assumption (*P* = .001). Thus, a user‐written command (strmst2) was used to compute restricted mean survival time (RMST) after a 6‐month follow‐up period and test the difference in restricted means between the randomized arms as an alternative measure of the intervention effect. RMST estimation procedure is robust and provides a meaningful interpretation of the treatment effect even when the proportional hazard assumption is violated (Cronin et al., 2016; Royston & Parmar, 2013). In all regression analyses, bootstrapping (with 1,000 iterations) was used to adjust for standard errors to account for the small number of clusters and obtain bias‐corrected CIs. Results were considered statistically significant at *P* < .05.

### 2.10 Ethical considerations

Ethical approval for this study was granted by the Kenya Medical Research Institute (KEMRI) Scientific and Ethical Review Unit (KEMRI/RES/7/3/1‐NON‐SSC/443). Participants provided informed consent after being provided with information and full disclosure regarding the study.

## 3 RESULTS

The study flow diagram is presented in Figure 1. A total of 901 pregnant women and their respective children were assessed for eligibility, 823 were enrolled in the study, randomized and followed up, and 579 participants were included in the analysis (Figure 1). A comparison of the characteristics of the subjects included in the analysis, and those lost to follow‐up showed no statistically significant differences (Table S1).

Socio‐demographic characteristics of study participants are presented in Table 3. There were no significant differences in socio‐demographic characteristics between the control and intervention groups at baseline. Imputed and non‐imputed proportions of exclusively breastfed children at different ages during the first 6 months are presented in Table 4. A large proportion of children in both the control (81.5%) and intervention (89.2%) group were exclusively breastfed during the first 2 months after birth. Between 3 and 6 months, although children in the intervention group were more likely to be exclusively breastfed than children in the control group, this difference was not statistically significant (84.6% vs. 67.1%; *P* = .072). Overall, children in the intervention group were more likely to be exclusively breastfed during the first 6 months than those in the control group (79.2% vs. 54.5%; *P* < .05). The OR (95% CI) for EBF were 1.88 (0.85, 2.90), for children aged less than 3 months, and 2.69 (1.54, 3.85) for children aged 3–6 months. Overall, mothers in the intervention group had about threefold increased odds of EBF for 6 months than mothers in the control group (OR 2.95; 95% CI 1.78, 4.10; *P* = .029) as shown in Table 4.

**TABLE 3:** Baseline socio‐demographic characteristics (*N* = 823)

Maternal characteristics	Control (*N* = 472)		Intervention (*N* = 351)	
	*n*	%	*n*	%
Age in years				
14–20	82	17.3	42	12.0
21–24	123	26.0	83	23.7
≥25	268	56.7	225	64.3
Marital status				
Married	358	75.7	285	81.4
Not married	115	24.3	65	18.6
Education				
Less than primary	77	16.3	53	15.1
Primary	224	47.4	115	32.9
Post primary	172	36.4	182	52.0
Religion				
Christian	445	94.1	345	98.6
Non‐Christian	28	5.9	5	1.4
Occupation				
Formal employment	101	21.4	67	19.1
Informal employment	130	27.5	95	27.1
Unemployed	242	51.2	188	53.7
Parity				
0	196	41.4	119	34.0
1	120	25.4	81	23.1
>1	157	33.2	150	42.9
Household food security				
Secure	322	68.1	242	69.1
Moderate	102	21.6	82	23.4
Severe	49	10.4	26	7.4

*Note*: Adjusted for clustering.

**TABLE 4:** The proportion of children exclusively breastfed and the odds ratios for exclusive breastfeeding stratified by child's age

	Age (months)	Exclusively breastfed	Control *n* (%)	Intervention *n* (%)	Odds ratio [95% CI]	Intracluster coefficient
Complete‐case analysis						
	0–2 (*n* = 484)	No	50 (18.4)	16 (7.6)	1	
		Yes	222 (81.6)	196 (92.4)	2.76 [0.81, 4.71]	0.205
	3–6 (*n* = 426)	No	57 (25.8)	31 (15.1)	1	
		Yes	164 (74.2)	174 (84.9)	1.95 [0.93, 2.97]	0.339
	0–6 (*n* = 336)	No	67 (38.1)	26 (16.3)	1	
		Yes	109 (61.9)	134 (83.7)	3.17 [1.42, 4.92]	0.279
Analysis after imputation (*n* = 579)						
	0–2	No	59 (18.5)	28 (10.8)	1	
		Yes	260 (81.5)	232 (89.2)	1.90 [0.86, 2.90]	0.108
	3–6	No	105 (32.9)	40 (15.4)	1	
		Yes	214 (67.1)	220 (84.6)	2.70 [1.54, 3.85]	0.205
	0–6	No	145 (45.5)	54 (20.8)	1	
		Yes	174 (54.5)	206 (79.2)	2.95 [1.79, 4.10]	0.114

*Note*: The 95% CIs are based on bootstrapped standard errors to account for few clusters.

**FIGURE 2:**
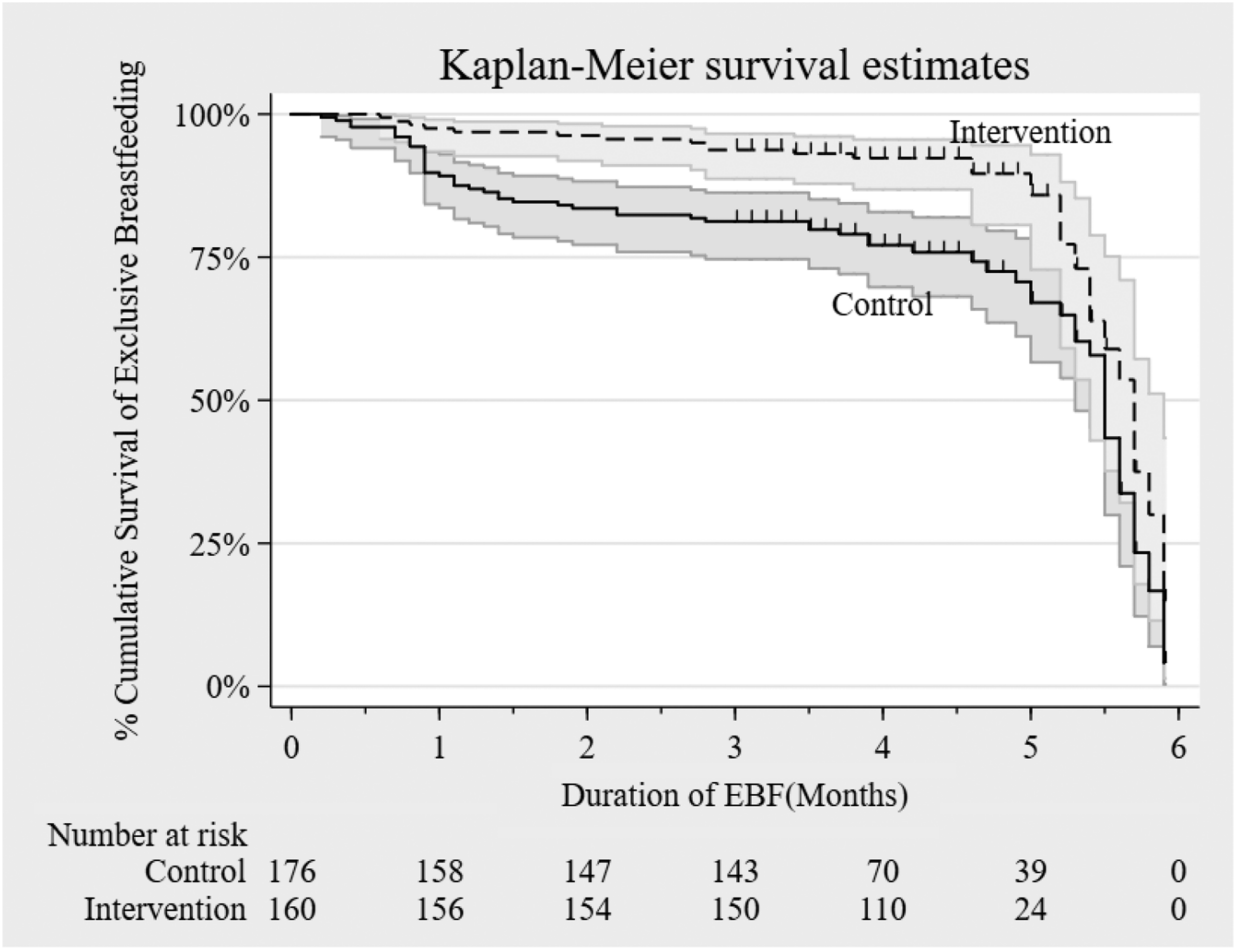
Kaplan–Meier survival estimates for stopping exclusive breastfeeding

Figure 2 is a Kaplan–Meier curve showing the probability of stopping EBF before 6 months by study group. Mothers in the control group had a higher probability of stopping EBF earlier than those in the intervention group (log rank test for population survival time *P* < .001). This result remained significant after multiple imputation (Figure S1).

The RMST estimates for the control and treatment arms were 4.64 and 5.32 months, respectively, before imputation. The RMST difference (95% CI) between the two groups was 0.69 (0.35, 1.03; *P* < .001) months, meaning children in the intervention group were, on average, exclusively breastfed 0.69 months (about 21 days) longer than those in the control group. After imputation, the RMST estimates for the control and intervention groups were 4.62 and 5.24 months, difference (95% CI) 0.62 (0.38, 0.85; *P* < .001), meaning children in the intervention group were breastfed 0.62 months (about 19 days) longer than children in the control group. The intracluster correlation coefficient was 0.027, meaning the variation within the clusters composed 2.7% of the total variation in the outcome. Overall, the median (interquartile range) EBF duration was 5.7 months (5.3, 5.9) in the intervention and 5.5 months (4.6, 5.7) in the control group before imputation. After imputation, the median duration was 5.7 months (5.1, 5.9) in the intervention and 5.2 months (3.8, 5.6) in the control group.

## 4 DISCUSSION

This study aimed to assess the effectiveness of the BFCI on EBF in a rural setting in Kenya. Findings from this study show that mothers in the intervention group were not only more likely to exclusively breastfeed their infants during the first 6 months of life, but they also exclusively breastfed them for a longer period than those in the control group, indications that the intervention was effective. Such improvements in EBF are important for child survival given that suboptimal breastfeeding practices account for 11.6% of child deaths (Black et al., 2013). They are also likely to lead to significant economic gains as investment towards improving breastfeeding practices is estimated to result in U.S. $35 of economic return per dollar invested (WHO & UNICEF, 2019).

Similar findings have been reported by other studies (Coutinho et al., 2005; Kavle et al., 2019). In Brazil, a randomized trial, which aimed to compare the differences in the effectiveness of the BFHI and BFHI combined with home visits (similar in many ways to the BFCI but lacking community and mother support groups), showed that during the first 6 months, mothers who received home visits had a significantly higher EBF prevalence than mothers who did not (45% vs. 13%) (Coutinho et al., 2005). Similarly, a systematic review of 195 studies, which aimed to assess the effectiveness of breastfeeding interventions delivered in health systems and services, home and family environment, community environment, work environment, policy environment or combination of settings, showed that interventions delivered either in health settings or in the family/home environment increased EBF by more than 45%. Furthermore, interventions delivered in both health systems and the community led to a 152% increase in EBF (Sinha et al., 2015). This is an indication that continued support at home and in the community is required to promote EBF as demonstrated by our findings.

In Kenya, a nonrandomized study assessing implementation of the BFCI in Migori, using a multilevel integrated approach, showed that the BFCI led to a significant increase in EBF between 0 and 6 months (from 75.2% to 92.3%) in Migori county during the implementation period (Kavle et al., 2019). Our randomized cluster control study further strengthens this evidence to demonstrate that an intervention incorporated in the existing health system, using minimal resources, can lead to increased EBF rates as shown by higher EBF rates in the intervention group (79.2% intervention vs. 54.5% control).

The intervention was designed to work within the existing health system, which meant minimal resources were required for implementation. Implementation involved training of CHVs and CHEWs. CHVs were also required to visit mothers in their homes to share information about MIYCN, care and stimulation. These visits were complimented with bimonthly baby‐friendly meetings with CMSG members. In addition, CHVs had monthly support meetings with CHEWs and quarterly support supervisory visits by the SCHMT (Kimani‐Murage et al., 2015; Ministry of Health, 2016).

CHVs played a vital role in the success of this intervention. To ensure sustainability, there is a need for innovative solutions such as the incorporation of income‐generating activities (IGA) for the CHVs. For example, in this intervention, CHVs in each cluster received a $1,000 seed grant, which enabled them to generate income‐generating financial groups. The IGAs ranged from table banking, which is a form of pooling money together and lending back to group members at a favourable interest rate, to agri‐business activities such as farming and animal rearing. This resulted in the economic empowerment of the CHVs. Identification of key champions who will promote breastfeeding at the community level has also been identified as a potential strategy, which is likely to promote sustainability of the BFCI (Kavle et al., 2019). Active involvement of the SCHMT was vital especially when it came to support supervision of CHVs.

The CMSGs were a unique model that involved other community members including spouses, opinion and administrative leaders and health professionals in providing support to the mothers. The biggest hindrance to setting up and sustaining the activities of these support groups was a lack of tangible incentives. However, in cases where the CMSGs were established and running, participants—especially mothers in the group—came together to establish their own IGAs, strengthening the peer support they offered to each other.

### 4.1 Strengths and limitations

We used a cluster randomized controlled design, which provides the highest level of evidence to answer the research question, and this is the first time this type of design has been used to test the BFCI in the Kenyan context. The community‐based structure, which included active involvement of families and the community, ensured mothers were supported and encouraged to exclusively breastfeed. We also had assured continuum of care because of strong linkages between the community and health facilities. Nonetheless, this study has some limitations. First, a relatively large proportion of the population was lost to follow‐up, which may have created selection bias. However, a comparison of the characteristics of subjects excluded from the study and those included revealed no statistically significant differences. Secondly, this study included a small number of clusters, with less than 10 clusters per treatment arm. To mitigate this limitation, we used bootstrapping to derive bias‐adjusted standard errors and hence CIs. Lastly, although dietary information was collected 24‐h and 3‐day recalls, the same outcome measure was used at each time point across all groups; the comparisons made are therefore valid.

## 5 CONCLUSION

This study showed that implementation of the BFCI within the existing community health structure has the potential to increase EBF rates in rural locations in Kenya. The BFCI's structure, which relies only on the existing health system to train and support CHVs as well as the setting up of community and mother support groups, means that it has good potential for scaling beyond Koibatek to other rural areas of Kenya. An obstacle to scaling, which could exist, relates to the need for CHVs to be compensated for their work. In this study, we provided a small‐scale start‐up resource for IGAs to support the programme locally at the request of the community. Further research is needed to understand how this aspect of the programme could be scaled.

## CONFLICTS OF INTEREST

The authors declare that they have no conflicts of interest.

## CONTRIBUTIONS

EWK‐M and JK, co‐principal investigators, provided overall leadership to study; ANM, joint first author, led drafting of the manuscript; EWK‐M, JK, NM, STM and PG conceptualized, designed and implemented the study. BS, FW, PM supervised and coordinated data collection and intervention implementation; PG and EWK‐M provided guidance on analysis plan; BMM analysed data with support from CW, EWK‐M, PG and ANM; CW, FW, PM, EWK‐M and PG reviewed subsequent drafts. All authors reviewed and approved the final manuscript.

## Supporting information


**Figure S1:** Kaplan–Meier survival estimates for stopping exclusive breastfeeding after multiple imputation

**Table S1:** A comparison of the maternal characteristic of children included in the study and those excluded

## Data Availability

The data that support the findings of this study are available upon request from the African Population and Health Research Center, Microdata portal (Kimani‐Murage et al., 2017).
