# Supplementary material for: Effectiveness of the baby‐friendly community initiative on exclusive breastfeeding in Kenya
Source: Matern Child Nutr. 2021 Feb 2;17(3):e13142. doi: 10.1111/mcn.13142 (PMC8189218; doi:10.1111/mcn.13142)
Supplement: Supplementary file 1 — Figure S1: Kaplan–Meier survival estimates for stopping exclusive breastfeeding after multiple imputation [file MCN-17-e13142-s002.docx]

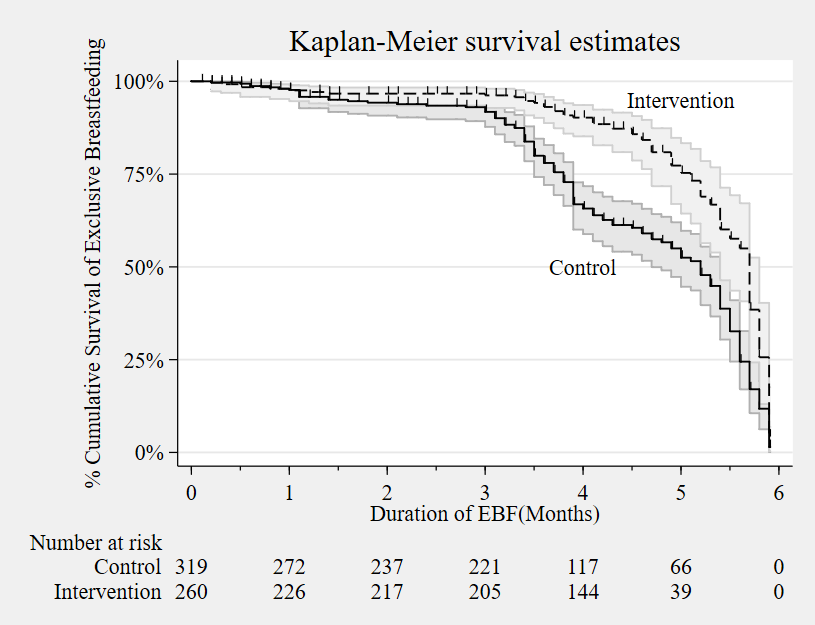


Supplementary Figure 1: Kaplan-Meier survival estimates for stopping exclusive breastfeeding after multiple imputation
