# Supplementary material for: Effectiveness of the baby‐friendly community initiative on exclusive breastfeeding in Kenya
Source: Matern Child Nutr. 2021 Feb 2;17(3):e13142. doi: 10.1111/mcn.13142 (PMC8189218; doi:10.1111/mcn.13142)
Supplement: Supplementary file 2 — Table S1: A comparison of the maternal characteristic of children included in the study and those excluded [file MCN-17-e13142-s001.docx]

Supplementary Table 1: A comparison of the maternal characteristic of children included in the study and those excluded

| **Characteristics** | | **Control n (%)** | | | **Intervention n (%)** | | | **Total (intervention + control) n (%)** | | |
| --- | --- | --- | --- | --- | --- | --- | --- | --- | --- | --- |
|  |  | **Included (n=319)** | **Excluded (n=154)** | **P-value** | **Included (n=260)** | **Excluded (n=90)** | **P-value** | **Included (n=579)** | **Excluded (n=244)** | **P-value** |
| Mothers age in years | |  |  |  |  |  |  |  |  |  |
|  | 14 - 20 | 50 (15.7) | 32 (20.8) | 0.369 | 30 (11.5) | 12 (13.3) | 0.652 | 80 (13.8) | 44 (18.0) | 0.261 |
|  | 21 - 24 | 75 (23.5) | 48 (31.2) | 0.128 | 57 (21.9) | 26 (28.9) | 0.181 | 132 (22.8) | 74 (30.3) | 0.023 |
|  | 25+ | 194 (60.8) | 74 (48.1) | 0.198 | 173 (66.5) | 52 (57.8) | 0.135 | 367 (63.4) | 126 (51.6) | 0.060 |
| Marital Status | |  |  |  |  |  |  |  |  |  |
|  | Married | 245 (76.8) | 113 (73.4) | 0.653 | 215 (82.7) | 70 (77.8) | 0.319 | 460 (79.4) | 183 (75.0) | 0.373 |
|  | Not Married | 74 (23.2) | 41 (26.6) |  | 45 (17.3) | 20 (22.2) |  | 119 (20.6) | 61 (25.0) |  |
| Highest education level | |  |  |  |  |  |  |  |  |  |
|  | Less Than Primary | 49 (15.4) | 28 (18.2) | 0.776 | 37 (14.2) | 16 (17.8) | 0.591 | 86 (14.9) | 44 (18.0) | 0.602 |
|  | Primary | 155 (48.6) | 69 (44.8) | 0.461 | 85 (32.7) | 30 (33.3) | 0.954 | 240 (41.5) | 99 (40.6) | 0.901 |
|  | Post Primary | 115 (36.1) | 57 (37.0) | 0.940 | 138 (53.1) | 44 (48.9) | 0.756 | 253 (43.7) | 101 (41.4) | 0.822 |
| Occupation | |  |  |  |  |  |  |  |  |  |
|  | Formal Employment | 63 (19.7) | 38 (24.7) | 0.739 | 47 (18.1) | 20 (22.2) | 0.585 | 110 (19.0) | 58 (23.8) | 0.588 |
|  | Informal Employment | 86 (27.0) | 44 (28.6) | 0.927 | 66 (25.4) | 29 (32.2) | 0.597 | 152 (26.3) | 73 (29.9) | 0.741 |
|  | Unemployed | 170 (53.3) | 72 (46.8) | 0.760 | 147 (56.5) | 41 (45.6) | 0.444 | 317 (54.7) | 113 (46.3) | 0.526 |
| Parity | |  |  |  |  |  |  |  |  |  |
|  | Null | 121 (37.9) | 75 (48.7) | 0.189 | 85 (32.7) | 34 (37.8) | 0.696 | 206 (35.6) | 109 (44.7) | 0.216 |
|  | One | 84 (26.3) | 36 (23.4) | 0.489 | 60 (25.8) | 21 (23.3) | 0.962 | 144 (24.9) | 57 (23.4) | 0.645 |
|  | > 1 | 114 (35.7) | 43 (27.9) | 0.369 | 115 (44.2) | 35 (38.9) | 0.518 | 229 (39.6) | 78 (32.0) | 0.250 |
| Household food security | |  |  |  |  |  |  |  |  |  |
|  | High | 214 (67.1) | 108 (70.1) | 0.868 | 173 (66.5) | 69 (76.7) | 0.732 | 387 (66.8) | 177 (72.5) | 0.722 |
|  | Moderate | 69 (21.6) | 33 (21.4) | 0.985 | 67 (25.8) | 15 (16.7) | 0.692 | 136 (23.5) | 48 (19.7) | 0.735 |
|  | Severe | 36 (11.3) | 13 (8.4) | 0.865 | 20 (7.7) | 6 (6.7) | 0.903 | 56 (9.7) | 19 (7.8) | 0.851 |
